# Adoption of online mathematics learning in Ugandan government universities during the COVID-19 pandemic: pre-service teachers’ behavioural intention and challenges

**DOI:** 10.1007/s44217-023-00035-0

**Published:** 2023-04-13

**Authors:** Geofrey Kansiime, Marjorie Sarah Kabuye Batiibwe

**Affiliations:** 1 Department of Science Education, Faculty of Education, Kabale University, P. O. Box 317, Kabale, Uganda; 2 Department of Science, Technical and Vocational Education, School of Education, College of Education and External Studies, Makerere University, P. O. Box 7062, Kampala, Uganda

**Keywords:** Adoption of online learning, Behavioural intention, COVID-19, Kabale University, And Pandemic

## Abstract

In the wake of COVID-19, higher education institutions worldwide were forced to continue teaching and learning through online means. However, it was only during the pandemic that institutions in Uganda, such as Kabale University, embraced online learning. Against this background, one could not predict how students drastically adapted to the new normal, especially in mathematics, which requires a lot of practice. Thus, this study sought to establish the relationship between behavioural intention to use technology and the adoption of online mathematics learning among pre-service teachers at Kabale University. We conceptualized behavioural intention to use technology according to the Unified Theory of Acceptance and Use of Technology (UTAUT) as comprising four factors: performance expectancy, effort expectancy, facilitating conditions, and social influence. This mixed methods study followed a cross-sectional correlational survey and hermeneutic phenomenological research designs. We collected data from 140 pre-service mathematics teachers, who were sampled using stratified and simple random sampling techniques, through a self-administered questionnaire. Also, we collected qualitative data through nine face-to-face interviews of pre-service mathematics teachers using criterion sampling, where the most prominent criterion was the participant's experience with the phenomenon under study. Using Pearson’s linear correlation, results showed that all UTAUT constructs were related to the adoption of online learning. Simple linear regression revealed that facilitating conditions were the strongest predictor. Furthermore, the narrative analysis indicated that, among others, a lack of technological knowledge hindered learners’ effective participation in online mathematics lectures. Therefore they barely benefited from online learning. Thus, we recommend government universities enhance teachers’ and learners’ technological knowledge, among other facilitating conditions such as establishing strong on-campus Wi-Fi connections as online learning continues.

## Introduction

In this post-COVID-19 pandemic era, online learning has become inescapable worldwide. Online learning is where facilitators deliver course content in a virtual space and fully use all learning materials and media [48]. Thus, online learning courses help learners to achieve their educational objectives and further support on-the-job learning by providing diverse communication tools and flexible learning options [51]. Based on this milieu, devices such as radios and televisions, as well as newer digital technologies, such as computers and the Internet, have been seen as potentially powerful enabling tools for this educational change and reform [59]. Besides, information communications technology (ICT) facilitates distance education [53] in all curriculum subjects. For example, ICT improves how mathematics should be taught and enhances students’ understanding of basic concepts [35]. Moreover, if teachers regularly use ICT in mathematics classrooms at all levels of education, learners will ultimately learn the subject better [49]. In addition, the learners will be able to use ICT appropriately, skilfully, and efficiently to do mathematics in technology-rich environments where they will study and work.

Contextually, Uganda has experienced growth in its ICT sector over the past decade due to the liberalization of ICT's acquisition, use, and application [55]. The country has also realized a similar trend in other sectors, such as health, industry, and agriculture, but not education [31]. Following the COVID-19 pandemic in early 2020, higher education institutions (HEIs) worldwide closed. During such unprecedented times, teaching and learning took place remotely through online processes [38]. However, most HEIs in most countries still needed to prepare for this new normal [27]. Emphasizing Uganda, HEIs struggled to continue teaching and learning online as it was not a common practice. Some universities had learning management systems (LMSs) in place, for example, Makerere University E-Learning Environment (MUELE) at Makerere University, Kyambogo EKampus Learning Management System (KELMS) at Kyambogo University, and Moodle-based LMS at Makerere University Business School. However, the learners and staff seldom embraced them before the pandemic [11].

Of all the nine government universities in Uganda, namely Makerere, Kyambogo, Kabale, Soroti, Lira, Gulu, Busitema, Muni universities, and Mbarara University of Science and Technology, Kabale University embraced online learning for the first time [50] during the COVID-19 lockdown through its newly acquired Moodle-based LMS. Against this background, one could not predict how learners at this university drastically adapted to online learning, especially in mathematics, a subject which requires a lot of practice. Therefore, we sought to understand the pre-service mathematics teachers’ behavioural intention to use technology and how these related to their adoption of online mathematics learning. In understanding behavioural intention to use technology in learning mathematics online, we considered the Unified Theory of Acceptance and Use of Technology (UTAUT) framework developed by Venkatesh, Morris, Davis, and Davis [61]. They argued that the UTAUT framework identifies four key factors: performance expectancy, effort expectancy, social influence, and facilitating conditions as vital predictors of behavioural intention to use technology and the actual use of technology, primarily in organizational contexts.

While performance expectancy is the degree to which technology benefits users when performing certain activities [28], effort expectancy is the level of comfort associated with using technology [61]. Meanwhile, social influence is the degree to which the user perceives that significant others believe they should use technology [61], and facilitating conditions is the degree to which a person believes that the existing organizational and technical infrastructure can support the use of technology [5]. Having conceptualized behavioural intention to use technology as the four constructs of the UTAUT framework, this study sought to establish the relationship between each of these constructs and the adoption of online mathematics learning among pre-service teachers at Kabale University. In particular, our specific research objectives were to establish the relationship between:

- Performance expectancy and the adoption of online mathematics learning
- Effort expectancy and the adoption of online mathematics learning
- Social influence and the adoption of online mathematics learning
- Facilitating conditions and the adoption of online mathematics learning

### Literature review

**Fig. 1:**
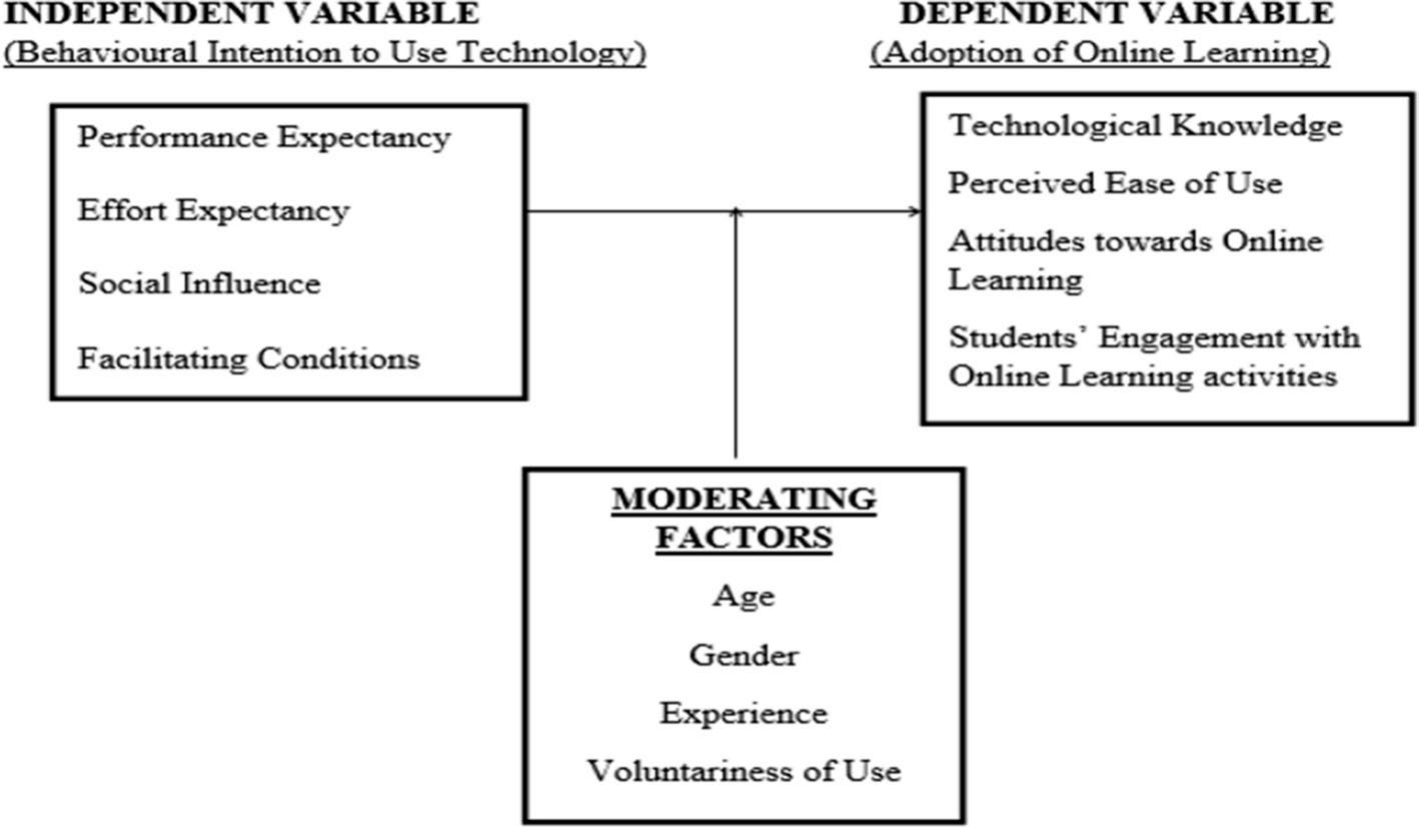
Conceptual framework of the study variables

In this section, we present the conceptual framework that links behavioural intention to use technology and the adoption of online learning in Fig. 1 and the literature related to each study objective.

From Fig. 1 as shown, the behavioural intention to use technology was conceptualized according to the UTAUT framework and adoption of online learning as technological knowledge (TK), perceived ease of use of the technology (PEU), student’s attitude towards online learning(ATOL), and students’ engagement in online learning activities (EOL). Although according to the UTAUT framework, age, gender, experience, and voluntariness of use affect the adoption of online learning, these were kept constant in this study for the fact that respondents were within the same age bracket, had no prior experience with online learning, and online learning was mandatory where respondents had no choice and therefore for these reasons, gender too was kept constant.

### Performance expectancy and the adoption of online learning

Online instruction in mathematics learning enhances students’ interactions because they display positive engagement with remote learning experiences [19]. For example, learning mathematics using mobile phones helped students to learn through collaboration and teamwork [13]. Meanwhile, students’ resilience in mathematics was always high during online learning because it positively influenced their mathematical reasoning and communication skills [6, 26]. Besides, online mathematics learning activities made students gain more interest, understanding, and confidence in the subject [62]. This finding was also portrayed by Tay, Lee, and Ramachandran [56] when they asserted that students’ engagement in online learning contexts was paramount to their mathematics learning.

Thus, it is evident that many scholars, such as Moreno-Guerrero, Aznar-Díaz, Cáceres-Reche, and Alonso-García [47], insist that online learning methods positively influence autonomy, participation, mastery of mathematical concepts, and improvement in results and grades. Similarly, according to Almarashdi and Jarrah [3], distance learning provides flexibility in choosing the time to perform and deliver tasks, even at night. On the contrary, online learning leads learners to lack opportunities to learn mathematics with and from their peers [38]. This argument could imply that the benefits of online learning vary with the kind of technology selected for use. Thus, in the context of Uganda, a developing country, we sought to test the following hypothesis, hypothesis One.

H_01_: There is no relationship between performance expectancy and the adoption of online mathematics learning.

### Effort expectancy and the adoption of online learning

Effort expectancy is related to technology use [4]. This phenomenon has been widely studied, and different scholars have given its merits and demerits. For instance, Ariyanti and Santoso [9] found that students’ average mathematics learning outcomes and responses towards the subject after online learning proved to be more significant than those before online learning, which they attributed to the ease of use of technology by the students, hence that positive change. In dissent, Mensah [46] found that mathematics tutors used ICT for general applications such as sending emails but rarely used computer-based technology. They hardly integrated ICT into teaching and learning mathematics because they found it challenging.

Furthermore, it was observed that students asked or answered questions with knowledge during online mathematics learning. However, they did not continue with further questioning when they encountered difficulties in using technology to solve problems [34]. Thus, one of the barriers to using digital technologies in higher education classrooms is the instructors' inability to support learners’ use of technology [12] due to their lack of knowledge or skills [39]. In affirmation, Agyei and Voogt [2] also indicated that several barriers, such as a lack of knowledge about integrating ICT in lessons and the different ICT tools available, were why mathematics teachers did not incorporate ICT in their instruction. Given such challenges, we sought to establish the following hypothesis, hypothesis Two, in the context of Uganda, a developing country.

H_02_: There is no relationship between effort expectancy and the adoption of online mathematics learning.

### Social influence and the adoption of online learning

Online teaching and learning should be devoted predominantly and purposefully to activities that promote student motivation and encouragement and provide opportunities for socialization [51]. However, Azmat and Ahmad [10] opined that the lack of social interaction was a global challenge to the effectiveness of online learning. They drew this observation from their study, where they found that a lack of social interaction affected learners in many ways. For example, it activated psychological issues such as depression, fear of loneliness, and boredom and further affected learners’ satisfaction levels. In agreement, Calder, Jafri, and Guo [18] also revealed that the inability to interact directly with other learners for clarification and mediation was the major frustration with online collaborations.

Moreover, such consequences have no boundaries. For instance, Marbán, Eqbal, Afnan, and Walaa [44] observed no significant differences in deploying social media platforms during mathematics learning based on gender, age group, and school type. This indication meant that social interaction during online teaching and learning affects all learners, regardless of gender, age, or school type.

Besides peers, parents also have educational responsibilities, which became crucial during the COVID-19 pandemic when teaching and learning completely shifted to an online mode [43]. Accordingly, learners needed support in accessing and completing online learning, especially those embracing online learning for the first time. Besides, parents also had to encourage their children by availing the required technology and financially supporting them by providing data bundles or paying for internet connection, among others [1]. For first-time users, Jenßen, Gierlinger, and Eilerts [36] indicated that perceived control of the ICT tools was much more critical than their perceived value in teaching and learning. Based on this claim, the learners needed their teachers’ support and therefore, in this study’s context, we sought to test the following hypothesis, hypothesis Three.

H_03_: There is no relationship between social influence and the adoption of online mathematics learning.

### Facilitating conditions and the adoption of online learning

Facilitating conditions are related to sufficient resources and support for individuals to use technology [28, 61]. Thus, in their absence, the adoption of online learning would be impeded. Various scholars have shown the facilitating conditions that inhibited or enhanced online learning. For example, internet connections and power interruptions are one of the most problematic aspects for students during online learning [17]. Furthermore and specifically, the absence of practical activities, inadequate support for structural exercises, few open resources, and insufficient access to online resources are significant challenges to online mathematics learning [52]. In addition, poor infrastructure, inadequate technology, and lack of sufficient technological tools affect the adoption of online learning [33].

On their part, Komuhangi, Mpirirwe, Robert, Githinji, and Nanyonga [41] noted that it always does not have to do with the availability of technology. Nevertheless, in some cases, teachers need to gain the knowledge to use ICT tools, the necessary skills to develop online courses, confidence in using ICT tools, and prior experience in e-learning. For instance, Tran, Phan, van Le and Nguyen [60] explored whether pre-service mathematics teachers at pedagogical universities in Vietnam could apply ICT integration as a supporting tool to develop their ICT competencies. However, they found that the teacher trainers' ICT integration during training pre-service teachers still needed to be improved, despite the availability of technologies. Moreover, other factors such as ineffective teacher professional development [21], low teacher self-efficacy [58], and teacher perceptions [40] also inhibit the adoption of online learning. Based on such empirical evidence, we sought to test the following hypothesis, hypothesis Four, in the context of Uganda, a developing country.

H_04_: There is no relationship between facilitating conditions and the adoption of online mathematics learning.

From the literature reviewed in this article, it is evident that performance expectancy, effort expectancy, social influence, and facilitating conditions are strong determinants of the actual adoption of online learning. Moreover, the literature reviewed in this article reveals that different scholars have given each determinant's importance, effects, constructs, impact, and casual relationships with the actual adoption of online learning, however, these have been in the contexts of other countries than developing ones such as Uganda. Notably, this study intended to establish if such findings from the literature were consistent in the context of Uganda, a developing country, especially since online learning was a new phenomenon in government universities.

## Methods

### Research approach and design

We used a mixed-methods approach [37] for this study. Further, we adopted a cross-sectional correlational survey and hermeneutic phenomenological research designs [22, 23]. We chose a correlational design due to our interest in establishing the relationship between pre-service teachers’ behavioural intention to use technology and their actual adoption of online mathematics learning. The survey design was the effect that we collected data from a large sample and the cross-sectional design because we collected them at one given time, once and for all, to make inferences about the population of our interest. Meanwhile, we selected the hermeneutic phenomenological design due to our interest in sharing the pre-service teachers’ personal experiences regarding online mathematics learning through conversational interviews.

### Population and participants

At the time of this study, Kabale University had a total of 205 pre-service mathematics teachers, of which 77, 71, and 57 were in Years One, Two, and Three, respectively. Thus, these constituted the study population. Of the 205 pre-service mathematics teachers, 140, which number we determined from Krejcie and Morgan’s [42] table for sample size determination, participated in this study. This sample size was the minimum sample size at the 95% confidence interval recommended for the population size. While Table 1 reveals the demographic variables of the survey participants, Table 2 presents the characteristics of those interviewed.

**Table 1:** Distribution of survey participants by demographic characteristics

Variables	Categories	Frequency	Percentage (%)
Sex of respondent	Male	101	72.1
	Female	39	27.9
Year of study	First-year	53	37.9
	Second-year	48	34.3
	Third-year	39	27.9
Mathematics lectures had online	0–5	62	44.3
	6–10	43	30.7
	Above 10	35	25.0
Had you used online learning websites for learning before COVID-19?	Yes	7	5.0
	No	133	95.0
Which websites do you use for learning mathematics?	Zoom	2	1.4
	Google meet	55	39.3
	Big blue button	4	2.9
	Others	1	0.7
	Any two	21	15.0
	All the three	57	40.7

**Table 2:** Characteristics of the interview participants

No	Name	Gender	Year of study	Number of online mathematics lectures	Age
1	Irene	Female	I	06	20
2	Peter	Male	I	11	22
3	Martin	Male	I	09	21
4	Sarah	Female	II	07	21
5	Joel	Male	II	12	23
6	Hudson	Male	II	13	22
7	Jackie	Female	III	10	22
8	Richard	Male	III	08	24
9	Tom	Male	III	10	23

### Sampling strategies

The 140 participants were a combination of all the years of study, and to ensure that each year was well represented, we used proportionate stratified sampling [7] so that 53, 48, and 39 pre-service mathematics teachers of Years One, Two, and Three respectively participated in this study. Furthermore, to select these, we used simple random sampling in each year of study. Additionally, aside from the 140 participants, we conducted in-depth face-to-face interviews with nine pre-service mathematics teachers who were purposively sampled, three from each year of study. Although we intended to interview more, data saturation occurred.

### Data collection methods and instruments

We collected data through a survey and face-to-face interviews. Thus, we used a self-administered questionnaire (SAQ) and an interview guide. The adapted SAQ [15, 32, 45, 61] had three sections: Sections A on the demographic variables of participants, B on the participants’ behavioural intention to use technology, and C on the actual adoption of online mathematics learning. Section A had five items on the demographic characteristics of the participants based on a nominal scale. Sections B and C had 17 and 19 items, respectively, on ordinal scales, which we based on a five-point Likert scale where participants had to select from Strongly Disagree = 1, Disagree = 2, Undecided = 3, Agree = 4, and Strongly Agree = 5. The SAQ was valid since, when tested, it had a content validity index (CVI) of 0.96 [7]. The Cronbach reliability coefficients of TK, PEU, ATOL, EOL, PE, EE, SI, and FC were 0.750, 0.846, 0.771, 0.796, 0.577, 0.702, 0.558, and 0.706 respectively. For PE and SI, since their coefficients were below 0.7, exploratory factor analysis was conducted on them. PE items loaded on two factors, with three items loading on the first factor and one on the second. The items on the first factor had loadings of greater than 0.5 and therefore were considered valid. The item on the second factor was dropped. For SI, all four items loaded on one factor, but one item that had a loading of 0.413 was dropped. A confirmatory factor analysis was repeated on the valid items of PE and SI and Cronbach reliability coefficients of 0.793 and 0.709 were obtained respectively. Thus, the SAQ was considered reliable [24]. The interview guide had three open-ended questions inviting participants to share their challenges during online mathematics lectures, how they coped with those challenges, and their suggestions for improving online mathematics learning. These open-ended questions produced verbatim comments, which added depth and meaning [16] to the SAQ items.

### Data analysis

Quantitative data were analyzed using descriptive and inferential statistics [7]. At the univariate level, we computed frequencies and percentages of the demographic variables. Furthermore, we calculated frequencies, percentages, means, and standard deviations of items of each construct of behavioural intention and adoption of online mathematics learning. At the bivariate level, we tested the four hypotheses of this study using Pearson’s Linear Correlation Coefficient, before which we performed aggregate indices on each of the constructs of behavioural intention and adoption of online mathematics learning [57]. In addition, simple linear regression was performed at the multivariate level to determine how much performance expectancy, effort expectancy, social influence, and facilitating conditions explained the adoption of online mathematics learning. On the other hand, we analyzed qualitative data by categorizing them according to UTAUT constructs and then giving narratives within each category [20].

### Ethical considerations

We explained the purpose of the study to the participants, and all of them voluntarily agreed to participate by signing a consent form. However, the SAQ remained anonymous to ensure their confidentiality, and we used its responses for only research purposes. Furthermore, we protected the identity of the interviewees in this article by using pseudonyms such that no reader could match any response to an individual participant [30].

## Findings

### Adoption of online learning of mathematics

We assessed the participants' level of readiness and their perceptions towards the adoption of online mathematics learning using four factors, namely:—their technological knowledge (TK), perceived ease of use (PEU) of technological tools, attitude towards online learning (ATOL), and engagement with online learning (EOL). Table 3 presents the descriptive statistics, namely frequencies, percentages, means, and standard deviations of the participants’ responses to the items that measured their adoption of online mathematics learning.

**Table 3:** Descriptive statistics on the adoption of online learning of mathematics

	Item	SA count (%)	A count (%)	N count (%)	D count (%)	SD count (%)	Mean	Standard deviation
TK1	I can create web pages for learning mathematics	14 (10.0)	28 (20.0)	11 (7.9)	37 (26.4)	50 (35.7)	2.90	1.404
TK2	I can use social media apps like blogs and Facebook to learn mathematics	27 (19.3)	41 (29.3)	17 (12.1)	29 (20.7)	26 (18.6)	3.58	1.421
TK3	I can use collaborative apps like Google sites to learn mathematics	38 (27.1)	54 (38.6)	11 (7.9)	19 (13.6)	18 (12.9)	2.46	1.359
TK4	I can use apps for communication, like Voice threads and podcasts, to learn mathematics	27 (19.3)	48 (34.3)	22 (15.7)	16 (11.4)	27 (19.3)	2.77	1.401
TK5	When learning mathematics, I can use apps for online note-taking, like Diigo and Wall wisher	12 (8.6)	20 (14.3)	20 (14.3)	32 (22.9)	56 (40.0)	3.71	1.348
PEU1	Learning to operate a computer/ smartphone/ laptop for learning mathematics has been easy	33 (23.6)	49 (35.0)	21 (15.0)	25 (17.9)	12 (8.6)	2.53	1.266
PEU2	I find it easy to get a computer/smartphone/ laptop to learn mathematics online	26 (18.6)	36 (25.7)	20 (14.3)	32 (22.9)	26 (18.6)	2.97	1.409
PEU3	My interaction with a computer/ smartphone/ laptop during online mathematics learning is clear and understandable	19 (13.6)	44 (31.4)	23 (16.4)	35 (25.0)	19 (13.6)	2.94	1.288
PEU4	I find a computer/ smartphone/ laptop flexible to interact with during online mathematics learning	28 (20.0)	44 (31.4)	19 (13.6)	33 (23.6)	16 (11.4)	2.75	1.326
PEU5	I can quickly become skillful at using a computer/ smartphone/ laptop for online mathematics learning	31 (22.1)	59 (42.1)	20 (14.3)	21 (15.0)	9 (6.4)	2.41	1.175
PEU6	I find a computer/ smartphone/ laptop easy to use for online mathematics learning	22 (15.7)	45 (32.1)	26 (18.6)	26 (18.6)	21 (15.0)	2.85	1.314
ATOL1	I enjoy using technology devices such as computers/smartphones/ laptops for learning mathematics	35 (25.0)	40 (28.6)	15 (10.7)	28 (20.0)	22 (15.7)	2.73	1.434
ATOL2	I have a positive attitude towards using technology devices such as computers/ smartphones/laptops for learning mathematics	42 (30.0)	47 (33.6)	12 (8.6)	24 (17.1)	15 (10.7)	2.45	1.359
ATOL3	I am confident in using technology devices such as computers/ smartphones/laptops for learning mathematics	24 (17.1)	34 (24.3)	36 (25.7)	32 (22.9)	14 (10.0)	2.84	1.242
EOL1	I have fun during online learning of mathematics and discussions	21 (15.0)	43 (30.7)	24 (17.1)	30 (21.4)	22 (15.7)	2.92	1.325
EOL2	I participate actively in online learning of mathematics and discussions	14 (10.0)	36 (25.7)	32 (22.9)	30 (21.4)	28 (20.0)	3.16	1.288
EOL3	I help my classmates/course mates during online learning of mathematics	19 (13.6)	38 (27.1)	31 (22.1)	27 (19.3)	25 (17.9)	3.01	1.317
EOL4	I engage in online conversations during mathematics learning	25 (17.9)	50 (35.7)	20 (14.3)	24 (17.1)	21 (15.0)	2.76	1.340
EOL5	I post regularly in discussion forums during online learning of mathematics	14 (10.0)	40 (28.6)	25 (17.9)	31 (22.1)	30 (21.4)	3.16	1.323
	Overall mean						2.88	

From Table 3, the overall mean response of the participants on their adoption of online mathematics learning was 2.88. When rounded off to the nearest whole, this mean corresponds with code 3 on the five-point Likert scale, which indicates a neutral response. Thus, this confirms that pre-service mathematics teachers needed to be sure about their readiness to adopt learning mathematics online.

### Behavioural intention to use technology

The participants’ behavioural intention to use technology was conceptualized as their strength to use different technological tools such as ICTs in learning mathematics online and hence categorized according to UTAUT’s four constructs, namely:—performance expectancy (PE), effort expectancy (EE), social influence (SI), and facilitating conditions (FCs). The participants rated themselves on items that measured each of these four constructs, and we gave the results in the following subsequent subsections.

#### Performance expectancy

Table 4 presents the participants' descriptive statistics, namely:—frequencies, percentages, means, and standard deviations on their responses to the items that measured their expectancy of how the different technologies for learning mathematics online performed.

**Table 4:** Descriptive Statistics on Performance Expectancy

	Item	SA count (%)	A count (%)	N count (%)	D count (%)	SD count (%)	Mean	Standard deviation
PE1	Using online learning websites improves my learning of mathematics	16 (11.4)	33 (23.6)	16 (11.4)	38 (27.1)	37 (26.4)	3.34	1.387
PE2	Using online learning enhances my motivation to learn mathematics	21 (15.0)	39 (27.9)	19 (13.6)	28 (20.0)	33 (23.6)	3.09	1.424
PE3	Using online learning websites augments my performance in learning mathematics activities	9 (6.4)	34 (24.3)	16 (11.4)	38 (27.1)	43 (30.7)	3.51	1.322
PE4	I only find online learning websites for studying mathematics at my university	39 (27.9)	33 (23.6)	16 (11.4)	18 (12.9)	34 (24.3)	2.82	1.561
	Overall mean						3.19	

Table 4 reveals the participants’ overall mean response on the items that measured performance expectancy was 3.19. When rounded down, this mean corresponds with code 3 of the five-point Likert scale, which is neutral. This finding indicates that pre-service mathematics teachers were still determining whether technologies such as ICTs used to learn mathematics online during the pandemic exceeded their expectations regarding effective learning.

In line with the first objective of this study, we made the first hypothesis to the effect that:

1 $${\text{H}}_{{0{1}}} : {\text{There was no relationship between performance expectancy and the adoption of online learning of mathematics}}$$

To test hypothesis (1), we first generated aggregate indices on performance expectancy and adoption of online mathematics learning to make them numerical variables. To establish the relationship between these, we used Pearson’s Linear Correlation Coefficient (PLCC), whose coefficient, r, was 0.536, showing a positive linear relationship between performance expectancy and the adoption of online mathematics learning. However, given the size of the coefficient, this was of moderate strength. Furthermore, PLCC revealed a significant value/ p-value of 0.000, which was far less than α- the value of 0.05. Since p < α, the null hypothesis was rejected, implying that performance expectancy had a moderate, significant, and positive linear correlation with the adoption of online learning among pre-service mathematics teachers at Kabale University.

We first categorized interview responses according to UTAUT’s constructs. Within each category, we analyzed the narratives regarding pre-service mathematics teachers’ challenges with learning mathematics online during the pandemic, their coping strategies, and suggestions for improving online mathematics learning. Regarding performance expectancy, participants had difficulties receiving meaningful and instant feedback during online learning; following mathematical steps, calculation procedures, and explanations during online mathematics lectures; and insufficient concern by their lecturers regarding individual differences, especially in technological knowledge. For example, Irene stated, ‘*you find that you wanted to ask a lecturer about a step you had not understood, but he had already scrolled to another slide*.’ Nevertheless, Peter said, ‘*there were several occasions when I typed my questions in the chat box, but the lectures ended when they had not been responded to, and that was the end*.’ Further still, all interviewees maintained a lack of peer interactions during the online mathematics lectures and missed out on non-verbal communication from their lecturers, which they claimed meant a lot to them. In confirmation, Richard stated that ‘*during physical classes, it is easy to get encouraged by our lecturers’ facial expressions as opposed to the online lectures where you look at slide after slide*,’ which ‘*bored us too much*,’ said Tom.

Meanwhile, 40% of the interviewees reported getting more explanations from YouTube and reaching out to their lecturers, through phone calls and short message services for clarifications, over areas they had not grasped well as their coping strategies. However, 60% consulted with their peers mostly through their WhatsApp group fora. While Sarah was happy that ‘*sometimes lecturers offered offline support*,’ Martin contended that ‘*they did not have much time for such services, and it was only once in a while*.’ According to Joel, ‘*my classmates were my saviors because we always had mathematics discussions every after the weird and unrealistic lectures’*. As ways for improving online learning of mathematics during any pandemic reoccurrences, participants suggested that: universities should provide students with prior and thorough training in how to use and manipulate various technological tools for learning mathematics online before online learning commences; lecturers should record their lectures and send the video or audio clips to the students after the online lectures; and that while planning their lectures, lecturers should plan for a time during the lectures to respond to students’ questions, queries or misconceptions. These suggestions arose as a consequence of, for example, Jack’s claim that ‘*we were given a two-hour orientation on how to use Zoom and the newly introduced learning management system, Moodle, and before we could figure out what to do, lectures were already on*.’

#### Effort expectancy

We asked the participants to rate themselves on items that measured the effort they expected to invest in using online tools to learn mathematics during the pandemic. Table 5 shows descriptive statistics, namely:—frequencies, percentages, means, and standard deviations of their responses on the said items.

**Table 5:** Descriptive statistics on Effort Expectancy

	Item	SA count (%)	A count (%)	N count (%)	D count (%)	SD count (%)	Mean	Standard deviation
EE1	I find online learning tools easy to use during mathematics lectures	11 (7.9)	27 (19.3)	21 (15.0)	44 (31.4)	37 (26.4)	3.49	1.284
EE2	I can quickly become skilful at using online learning tools while studying mathematics	26 (18.6)	50 (35.7)	20 (14.3)	23 (16.4)	21 (14.3)	2.74	1.344
EE3	I am proficient at using online learning tools during my mathematics lectures	12 (8.6)	32 (22.9)	44 (31.4)	32 (22.9)	20 (14.3)	3.11	1.170
EE4	I will become proficient with time at using online tools for learning mathematics	52 (37.1)	54 (38.6)	16 (11.4)	9 (6.4)	9 (6.4)	2.06	1.152
EE5	Mathematics learning activities with online learning tools are clear and understandable to me	13 (9.3)	29 (20.7)	28 (20.0)	35 (25.0)	35 (25.0)	3.34	1.309
	Overall mean						2.95	

Table 5 indicates that the participants' overall mean response on the items that measured effort expectancy was 2.95, which corresponds with code 3 of the five-point Likert scale: neutral. This finding suggests that pre-service mathematics teachers were not yet sure of their expectations concerning the amount of effort required to use technological tools in learning mathematics online.

Corresponding to the second objective of this study, we sought to test the second hypothesis:

2 $${\text{H}}_{{0{2}}} : {\text{There was no relationship between effort expectancy and adoption of online learning of mathematics}}$$

Like earlier, we generated an aggregate index on effort expectancy to make it a numerical variable and went ahead to employ PLCC to establish whether a relationship between effort expectancy and adoption of online mathematics learning among pre-service teachers at Kabale University existed. We obtained a coefficient, r, equal to 0.663, showing a positive linear relationship between these variables but moderate strength. In addition, PLCC indicated a significant value/ p-value of 0.000, which was far less than α, the value of 0.005, and since p < α, the null hypothesis was rejected. Hence, effort expectancy and adoption of online mathematics learning had a significant positive linear correlation of moderate strength.

Regarding effort expectancy, all interviewees needed more skills to manipulate the technological tools effectively during online mathematics learning. This phenomenon resulted in diverting their attention from acquiring content knowledge to gaining self-directed technical knowledge proficiency in using the menus of the online learning tools. In affirmation, Hudson maintained that ‘*there was when I had burning questions, but I failed to get where to press to raise my hand*.’ Similarly, Martin recalled that ‘*there were many times lecturers posted links and attachments in the chat room, but because I did not know how to download them, I lost out*.’ However, he continued*, ‘since we were many, the lecturers could not make follow-ups.’* Nevertheless, according to 80% of the interviewees, they barely benefited from the online lectures due to the stiff competition between paying attention in class and manipulating online learning tools.

Findings indicate that 70% of the participants were demotivated by the effort required to learn mathematics online successfully. However, the remaining 30% revealed that their peers organized group discussions through video conferences, which they attended as a follow-up to the online lectures so that they could learn from those who had understood the mathematics concepts that had been taught earlier. This challenge was evident in Sarah’s response, who said that ‘*learning mathematics online was a nightmare for me, but I used to wait for those who had understood to discuss for me during our small group discussions after the lectures*.’ On his part, Joel claimed that ‘*I could not easily understand how Moodle works and so just gave up on it*.’ Correspondingly, ‘*because of the so many things I had to learn in a short time, like uploading work, using the chat box, and raising the hand in a Zoom lecture, I gave up and waited to learn from my colleagues*,’ said Jack.

In agreement, all interviewees requested that, if they existed, they should be introduced to digital tools and applications specifically designed for learning university mathematics and be made accessible to them. Such a suggestion descends from the interviewees’ experiences, such as, according to Richard, ‘*Zoom and Google Meet, for example, were not specific to only mathematics but communication tools for any learners’*. Similarly, Martin said that ‘*the lecturers uploaded their PowerPoint slides on Zoom, and it felt like a replacement of hard copy hand-outs in a physical lecture*.’ For Peter, ‘*talking mathematics procedures on Zoom was too superficial.'*

#### Social influence

The participants were required to rate themselves on items that measured how essential others think they should learn mathematics online. Table 6 shows descriptive statistics, namely:—frequencies, percentages, means, and standard deviations on their responses on items that measured social influence.

**Table 6:** Descriptive Statistics on Social Influence

	Item	SA count (%)	A count (%)	N count (%)	D count (%)	SD count (%)	Mean	Standard deviation
SI1	People who are important to me think I should use online learning tools to study mathematics	19 (13.6)	34 (24.3)	15 (10.7)	34 (24.3)	38 (27.1)	3.27	1.434
SI2	My peers and lecturers think I should use online learning tools to study mathematics	35 (25.0)	59 (42.1)	13 (9.3)	20 (14.3)	13 (9.3)	2.41	1.263
SI3	People who affect my learning behavior think I should use online learning websites when studying mathematics	25 (17.9)	35 (25.0)	21 (15.0)	29 (20.7)	30 (21.4)	3.03	1.429
SI4	Using online learning websites for learning mathematics is fashionable	43 (30.7)	38 (27.1)	21 (15.0)	13 (9.3)	25 (17.9)	2.56	1.460
	Overall mean						2.82	

The results in Table 6 indicate that the participants’ overall mean response on the items that measured the social influence of 2.82 corresponds with code 3 of the five-point Likert scale, which was neutral. This result shows that the pre-service mathematics teachers needed to figure out whether essential others, such as their universities, believed they should learn mathematics online.

Consistent with the third objective of this study, we sought to test the third hypothesis:

3 $${\text{H}}_{{0{3}}} : {\text{There was no relationship between social influence and adoption of online learning of mathematics}}$$

As has been the case for the other constructs, we generated an aggregate index on social influence to make it a numerical variable. We tested the relationship between the two variables in equation three using Pearson’s Linear Correlation Coefficient. The correlation coefficient, r, of 0.420, which we obtained, indicated a positive and linear relationship between social influence and the adoption of online mathematics learning. Given its size, however, it was of weak strength. Furthermore, we obtained a significant value/ p-value of 0.000, which was far less than α, of value 0.05, and since p < α, the null hypothesis was rejected. As a result, social influence and the adoption of online mathematics learning had a significant positive linear correlation which was weak in strength.

Regarding social influence, the interviewees were challenged by their parents’ economic statuses and the university’s and mathematics lecturers’ unpreparedness for online learning. According to the interviewees, many people lost their jobs during the pandemic. As a result, only a few parents would consistently provide data bundles for their children to keep up with online learning. Martin and Joel said they only attended online mathematics lectures when their parents could afford the much-needed data. Similarly, Tom maintained that ‘*although some telecommunication companies gave data subsidies, my single mother still could not meet the data expenses for me to attend daily lectures*.’

Nonetheless, Hudson believed that ‘*my parents knew the importance of attending online lectures, only that they could not afford the internet charges*.’ On his part, Peter insisted that ‘*the university was not ready for online learning but just picked up this arrangement because maybe it thought that its important since all other government universities had adopted it during the pandemic*.’ On the other hand, Sarah felt that ‘*lecturers seemed not prepared to teach online because some were fidgeting as much as we did*.’ According to her, ‘*some lecturers could not, for example, guide us on how to upload work or even make breakaway rooms on Zoom*.’

As a strategy for overcoming their challenges, interviewees pointed out that they got more explanations from their colleagues or other online applications such as YouTube and appropriate websites. They also reached out to their lecturers through phone calls, emails, or short message services for clarifications on the areas they needed to have grasped better. Peter recalled, ‘*I used to send some of my mathematics lecturers emails to request for conceptual support, and they used to suggest links and references for me for further reading*.’ Correspondingly, Irene stated,’ *my classmates were of much help because we used to discuss through video conferencing when MTN* [a telecommunication company] *provided data subsidies or bonuses*.’ Cognisant that the university, the mathematics lecturers, and parents believed that online learning was the perfect substitute for ordinary face-to-face lectures in unprecedented times such as the COVID-19 pandemic, the interviewees suggested that Kabale University should continue invigorating online learning. Further, it should vehemently orient and support mathematics lecturers in teaching online. In addition, they indicated that the university authorities should encourage parents to make every effort to support their children's online learning than looking at it as an economic punishment.

#### Facilitating conditions

The descriptive statistics, namely percentages, frequencies, means, and standard deviations in Table 7, indicate the participants’ responses on items that measured facilitating conditions.

**Table 7:** Descriptive Statistics on Facilitating Conditions

		SA count (%)	A count (%)	N count (%)	D count (%)	SD count (%)	Mean	Standard deviation
FC1	I have the resources for online learning websites while studying mathematics	18 (12.9)	39 (27.9)	19 (13.6)	27 (19.3)	37 (26.4)	3.19	1.422
FC2	It is necessary to use online learning websites while studying mathematics	22 (15.7)	46 (32.9)	25 (17.9)	31 (22.1)	16 (11.4)	2.81	1.269
FC3	Using online learning websites for mathematics fits well with how I like to learn	13 (9.3)	27 (19.3)	22 (15.7)	33 (23.6)	45 (32.1)	3.50	1.360
FC4	If I have problems using online learning websites while studying mathematics, I can solve them quickly	21 (15.0)	23 (16.4)	17 (12.1)	27 (19.3)	52 (37.1)	3.47	1.496
	Overall mean						3.24	

The results in Table 7 indicated an overall mean response on all the items that measured facilitating conditions of 3.24, which corresponded with code 3, neutral. This finding revealed that pre-service teachers needed more confidence in the organizational and technical infrastructure to support technology in learning mathematics.

Related to the fourth objective of this study, we sought to test the fourth hypothesis:

4 $${\text{H}}_{{0{4}}} : {\text{There was no relationship between facilitating conditions and adoption of online learning of mathematics}}$$

Further still, we generated an aggregate index on facilitating conditions to make it a numerical variable. We established the relationship between facilitating conditions and the adoption of online mathematics learning among pre-service teachers at Kabale University using Pearson’s Linear Correlation Coefficient. We obtained a correlation coefficient, r, of 0.654, showing a positive and linear relationship between the two variables of equation four. However, given the size of the coefficient, this was of moderate strength. In addition, we obtained a significant value/ p-value of 0.000, which was far less than α, the value of 0.05, and since p < α, the null hypothesis was rejected. Accordingly, facilitating conditions and adoption of online mathematics learning had a significant positive linear correlation of moderate strength.

Concerning facilitating conditions, the interviewees were challenged with their lack of technological knowledge, poor network/ internet connection, low bandwidth, high costs of ICT tools such as computers, laptops, or tablets to use in online learning, and inadequate financial resources by the university to facilitate effective online learning. For instance, Jack stated, ‘*the network continuously interrupted my lectures,’* and further that, *‘although there was Wi-Fi at the university, there was always slow or unstable connection due to many users.’* In addition, Irene maintained that ‘*me, I had a small ka* [tiny]*-smartphone and some apps’ views were compromised due to the limited window, yet, my guardian could afford to buy neither a laptop nor tablet for me*.’ However, to cope with such challenges, the interviewees underscored that they either bought their data in some cases or spotted Wi-Fi stable locations at the university premises to attend the online mathematics lectures regularly. Martin evidenced this assertion by saying, ‘*when I had an online mathematics lecture, I could travel very early to Makanga* [School of Medicine campus] *because their Wi-Fi was speedy and stable.’*

Considering the challenges the interviewees faced, they suggested that Kabale University should provide more computer laboratories commensurate with the number of students at the university. Since Kabale University is a government university, the government could loan students laptops as soon as they report to the university to ease online learning. Besides, they suggested that the university improve its network, internet connection, bandwidth, and Wi-Fi. Furthermore, the interviewees believed parents and guardians need sensitization about their expected roles, such as providing internet bundles for their children to facilitate their online learning. In affirmation, Joel insisted that ‘*probably the government could give laptops to every university student on loan and we pay when we start to work after graduating*.’ Relatedly, Sarah argued that the ‘*government should bargain with telecommunication companies to give us subsidized data bundles, or free internet connection to learning management systems [LMSs] and Zoom.* However, she continued, *‘our parents cannot afford data bundles on top of tuition fees.’*

To establish how much behavioural intention to use technology, perceived as performance expectancy, effort expectancy, social influence, and facilitating conditions, explained the adoption of online learning of mathematics, we performed a simple linear regression using equation five below:

5 $${\text{Adoption of Online Learning of Mathematics }} = x_{{1}} {\text{PE }} + x_{{2}} {\text{EE }} + x_{{3}} {\text{SI }} + x_{{4}} {\text{FC}}$$

In a statistical model, multicollinearity can lead to skewed results especially when determining how each independent variable effectively predicts the dependent variable. In this study, we verified multicollinearity by using the Variance Inflation Factor (VIF) to measure the strength of the correlation between the independent variables and the adoption of online learning in the simple linear regression analysis. Table 8 reveals that all VIF values for the relationships were below 5, meaning that all the correlations were of moderate strength, with no multicollinearity, and therefore included in the model. Further, simple linear regression revealed an adjusted R square (R^2^) of 0.561, which meant that behavioural intention to use technology, predicted as performance expectancy, effort expectancy, social influence, and facilitating conditions, explained 56.1% of the adoption of online mathematics learning. Thus, 56.1% is significant enough to justify the relationship between behavioural intention to use technology and the adoption of online mathematics learning among pre-service teachers at Kabale University. In addition, Table 8 shows the corresponding β coefficients that emerged from simple linear regression. These reveal the strength of each construct that predicts behavioural intention to use technology in explaining the adoption of online mathematics learning.

**Table 8:** Behavioural intention to use technology and the adoption of online learning of mathematics

Construct	Standardized beta (β)	Standard error	p-value	t-values	Effect size	VIF values
Facilitating conditions	0.326	0.061	0.000	4.368	0.86	1.765
Effort expectancy	0.316	0.076	0.000	4.029	0.89	1.948
Performance expectancy	0.180	0.060	0.010	2.623	0.64	1.491
Social influence	0.131	0.056	0.037	2.111	0.46	1.220

The significance level for the relationships is at α = 0.05

The results in Table 8 revealed that for every change in facilitating conditions, the adoption of online mathematics learning increased by 32.6% (β = 0.326). For every shift in effort expectancy, the adoption of online mathematics learning increased by 31.6% (β = 0.316). For every change in performance expectancy, the adoption of online mathematics learning increased by 18.0% (β = 0.180). For every change in social influence, the adoption of online mathematics learning increased by 13.1% (β = 0.131). Thus, the strongest predictor of behavioural intention was facilitating conditions, followed by effort expectancy, then performance expectancy, and trailed by social influence. Therefore, equation five becomes:

6 $${\text{Adoption of Online Learning of Mathematics}} = 0.{18}0{\text{PE}} + 0.{\text{316EE}} + 0.{\text{131SI}} + 0.{\text{326FC}}$$

Hence, facilitating conditions were the strongest predictor of the adoption of online mathematics learning among pre-service teachers at Kabale University. The coefficient of determination (R^2^) for the total variance explained by performance expectancy, effort expectancy, social influence, and facilitating conditions on the adoption of online learning in the model summary output was 0.574, meaning that 57.4% of the points should fall within the regression line. Meanwhile, all effect sizes are greater than 0.40 which means that all the relationships have practical significance. Since all the t-values are higher than 2, there’s greater confidence that behavioural intention to use technology is a predictor of the adoption of online learning.

### Discussion

The results of this study indicated that the participants were undecided regarding their self-rating on the adoption of online learning and their behavioural intention to use technology in terms of performance expectancy, effort expectancy, social influence, and facilitating conditions. These results are not surprising given that it was the first time for 95% of the participants to attend online learning. The participants’ indecision is a clear indicator that they are still determining how the technologies they use effectively perform and the effort it takes to successfully adopt their use during online learning. Furthermore, they are still deciding who else thinks the technologies they use for online learning are essential and beneficial. In addition, these participants are also still determining the conditions that should be in place to facilitate their online learning. In support of this finding, they revealed that they encountered several challenges, as mentioned in the previous section. However, such challenges are not unique to the context of this study, as several other scholars found similar challenges.

For instance, Mensah [46] observed that mathematics teachers rarely used computer-based technology and integrated ICT in teaching and learning mathematics because they needed help. Meanwhile, Kalogeropoulos, Roche, and Russo [38] observed that students could not learn mathematics with and from their peers during online learning. In addition, Harrell and Bynum [33] found poor infrastructure, inadequate technology, and a lack of sufficient technological tools as significant challenges that inhibited the successful adoption of online learning. Furthermore, Hidayah, Sa’dijah, Subanji, and Sudirman [34] observed that students did not continue questioning further during online mathematics learning when they encountered difficulties using technology [29] to solve problems. In Daghan’s [25] study, students considered using technology to learn online as a waste of time as they spent more time learning how to maneuver using the tools than learning the subject matter. In tandem, Jenßen, Gierlinger, and Eilerts [36] found that the students' perceived control over the technology used was much more important than its perceived value in teaching and learning. Besides, Bringula, Reguyal, Tan, and Ulfa [17] demonstrated that internet connections and power interruptions were the most problematic aspects of online education in the context of their study.

Concerning the relationship between behavioural intention to use technology and the adoption of online learning, this study revealed that each of the UTAUT constructs that predict behavioural intention to use technology: performance expectancy, effort expectancy, social influence, and facilitating conditions significantly and positively correlated linearly, however, with varying strengths. Remarkably, while social influence was of weak strength, performance expectancy, effort expectancy, and facilitating conditions had moderately strong [54] relationships. This finding confirms that they all determine whether a learner will or will not adopt online learning. This finding is consistent with global literature. For instance, if technology benefits users, they are highly likely to use it [28]. Different scholars, such as Dewi [26], have given the benefits of using technology in mathematics. In his study, he observed that online learning positively influenced students' mathematical reasoning and communication skills.

Further, students or teachers adopt online learning depending on the ease they associate with using technology [28]. Thus, the significant factors in students’ decision to participate in online courses and, therefore, their satisfaction with its use would include, among others: reduced logistic demands, increased learning flexibility, technology-enhanced learning, and reduced opportunity cost for getting an education, all of which result in higher student satisfaction [51]. This means that students get delighted with online courses once they get comfortable with using the selected technologies. Considering that 44.3% of the participants in this study attended fewer than five lectures for the entire year that mathematics was taught online, it is evident that they were dissatisfied with online learning probably because the technology used was not accessible or even available for them to use. Notably, people could only adopt online learning if the organizational and technical infrastructure supports technology use [5]. Accordingly, the availability of resources such as computers, widespread Internet access, easy access to mobile devices, and stable power support the adoption of online learning [14].

Although at the bivariate level, Pearson's Correlation Coefficient revealed that of the four UTAUT constructs, effort expectancy had the most substantial relationship with the adoption of online mathematics learning, at the multivariate level, simple linear regression indicated that the strongest predictor was facilitating conditions. However, looking at the difference in strength between effort expectancy (r = 0.663) and facilitating conditions (r = 0.654), it is marginal, and therefore both are strong predictors in the context of this study. Furthermore, simple linear regression revealed that assembled; all these four determinants explained 56.1% of pre-service mathematics teachers’ adoption of online learning. This finding implies that in as much as UTAUT constructs are strong predictors of the adoption of online learning, other factors account for 43.9% of the adoption of online learning.

The implication of these findings is that the time is now ripe for Kabale University and other higher education institutions in Uganda to strengthen online learning to tap into the potential advantages that come along with it. According to Appana [8], there are potential benefits of investing in online education, for example, increased access to knowledge, improved quality of learning, and better preparation of students for a knowledge-based society and lifelong learning opportunities. However, to realize these benefits, essential steps have to be taken, the first being training both teachers and students how to use various technologies because they are the actual academic organization that allows learning to take place [63]. According to the participants, they were trained twice, and online, before they started online learning. This training needed to be improved, given that they were handling the technology for the first time.

In addition, the various benefits of teaching and learning online using multiple technologies should be vividly explained to the trainees during training sessions. Moreover, since facilitating conditions were the strongest predictor of the adoption of online learning in this study, it suggests that Kabale University needs to invest more in organizational infrastructure and resources to enhance students’ adoption of online learning further. We base this suggestion on the participants’ submission that they were challenged with interrupted internet connections and scarce computers. We found out that Kabale University procured almost two hundred computer sets to kick-start online learning, and we note that this was an excellent beginning point. However, there is a need to ensure that these resources are used accordingly to avoid cases such as the one in Tran, Phan, Le, and Nguyen’s [60] study, in which they indicated that despite the availability of technologies, ICT integration in training pre-service teachers still needed to be improved.

### Conclusion and recommendations

The students’ behavioural intention to use technology significantly and positively relates to the adoption of online learning linearly. We conceptualized behavioural intention to use technology according to the four constructs of the UTAUT framework: performance expectancy, effort expectancy, social influence, and facilitating conditions. Of these, facilitating conditions were the strongest predictor of the pre-service teachers’ adoption of online mathematics learning. This finding implies that Kabale University has to invest more in resources and infrastructure to enhance online mathematics learning. This study has contributed to the existing literature by providing empirical evidence regarding the relationship between behavioural intention to use technology and the adoption of online learning in the context of a developing country. Thus, it has filled the gap that existed in the current literature as earlier indicated in Sect. 1.5. Further, in the context of this study, UTAUT was modified by keeping all its extraneous variables constant, given that the study dealt with a relatively homogenous population. This made the model unique in the context of this study. Despite the significant contribution of this study, it had limitations. For example, the analysis was applied to only Kabale University, one of the nine government universities. This means that these results can be generalized to Kabale University but not the other universities, especially that at least all of them had LMSs before the pandemic, save that they were not in active use.

In addition, we cannot guarantee that similar findings would be achieved if this study were replicated in private universities. Instead, we recommend that similar studies be conducted in government universities where LMSs existed before the pandemic and private universities. Moreover, comparative studies would be beneficial to policymakers and education agencies. Furthermore, since the study sample was 140, these results may only represent some of the pre-service mathematics teachers in Uganda. Besides, other studies could use a more significant sample. The constructs of UTAUT explained only 56.1% of the adoption of online learning of mathematics, meaning that other factors account for 43.9% of the adoption of online learning of mathematics. Thus, future research can be conducted to establish these, considering other theories, such as the Theory of Planned Behaviour. Lastly, since the study was predominantly quantitative, it could have limited an in-depth understanding of the phenomenon, and therefore future research will fill this gap.

## Data Availability

All data generated and analyzed during this study are included in this published article.
